# The rate of biliary adverse events in duct-to-duct living donor liver transplant compared with deceased donor liver transplant

**DOI:** 10.1016/j.igie.2023.10.010

**Published:** 2023-11-02

**Authors:** Pavlos Kaimakliotis, Karim T. Osman, Danitza Z. Lukac, Ali Shaat, Lina M. Nienaa, Nikola Natov, Mena Bakhit, Ann Marie Joyce, Amir A. Qamar

**Affiliations:** 1 Division of Gastroenterology, Lahey Hospital and Medical Center, Burlington, Massachusetts, USA; 2 Department of Internal Medicine, Lahey Hospital and Medical Center, Burlington, Massachusetts, USA; 3 Division of Transplantation and Hepatobiliary Diseases, Lahey Hospital and Medical Center, Burlington, Massachusetts, USA; 4 Department of Internal Medicine, Alexandria University, Alexandria, Egypt

## Abstract

**Background and aims:**

Living donor liver transplantation (LDLT) allows for decreased listing-to-transplant time in patients with end-stage liver disease but has been noted to be associated with higher rates of biliary adverse events. The aim of this study was to assess the adverse events of LDLT and compare them versus those of deceased donor liver transplantation (DDLT) patients.

**Methods:**

We retrospectively reviewed patients having undergone duct-to-duct anastomosis LDLT at a single center from 2011 to 2020. Exclusion criteria included pediatric patients and LDLT with Roux-en-Y hepaticojejunostomy. Patients were then matched 1:1 with DDLT. Matching was performed by age, gender, etiology of liver disease, and Model for End-Stage Liver Disease score. Outcomes of interest included incidence of biliary strictures, bile leak, stricture recurrence rate, and the number of interventions required for stricture resolution.

**Results:**

Fifty patients with LDLT were matched to 50 patients with DDLT. Bile leak occurred in 14 (38%) patients in the LDLT group versus 5 (10%) patients in the DDLT group (*P* = .001). Biliary strictures occurred in 14 (28%) versus 15 (30%) patients in the LDLT and DDLT groups, respectively (*P* = .68). There was no difference in the median (interquartile range) number of interventions required to resolve the strictures (3 [2-6] vs 4 [3-5]; *P* = .79]. Three patients in each group had recurrence of strictures after documented resolution. Mortality in both groups were similar (5 [10%] patients in the LDLT group vs 6 [12%] in the DDLT group; *P* = .75).

**Conclusions:**

Patients who underwent LDLT were equally as likely to develop anastomotic strictures compared with DDLT. Duct-to-duct anastomosis LDLT should be frequently considered in patients with end-stage liver disease with favorable anatomy.

Living donor liver transplantation (LDLT) is an excellent alternative to deceased donor liver transplantation (DDLT) given the long wait time for potential recipients and increased waiting list mortality.^1^ LDLT accounts for 5% to 7% of all liver transplants in the United States, although they are frequently clustered in high-volume centers.^2^ LDLT is becoming more common in the United States, with an increasing number of centers offering this option to patients.^3^

Despite the favorable outcomes associated with LDLT, including reducing time spent on the waiting list, biliary adverse events may occur at a higher rate of up to 40%.^4^^,^^5^ A more recent meta-analysis by Barbetta et al^6^ quoted a twofold higher risk of biliary adverse events with LDLT compared with DDLT. However, none of the studies in the subgroup was from the United States, and the type of anastomosis created was not indicated. It was also not stated whether these adverse events were bile leak or biliary stricture. Few studies from the United States have reported on specific biliary adverse events after LDLT, especially with a focus on anastomotic strictures.

The purpose of the current study was to assess biliary adverse events in LDLT at our institution compared with DDLT and add to the limited literature regarding outcomes of LDLT in the United States.

## Methods

This retrospective cohort study was conducted at a tertiary referral center for end-stage liver disease and liver transplantation (LT). Institutional review board approval was obtained before data collection (institutional review board #1719916-1). For this type of study, formal consent was not required. A total of 50 consecutive patients had undergone LDLT with duct-to-duct anastomosis from December 2011 to December 2020 at our institution. Exclusion criteria included pediatric patients and LDLT with Roux-en-Y biliary reconstruction.

### Matching

Patients were matched 1:1 with DDLT recipients. The DDLT recipients were obtained from a prospectively maintained list of patients who were being evaluated for LT at our institution. Authors were blinded to all variables, including biliary adverse events, recurrence of biliary strictures, and mortality. The only variables available to the authors before matching were the ones used for matching. Patients post-DDLT were matched with LDLT recipients by age (within 10 years’ range), gender, etiology of liver disease, and Model for End-Stage Liver Disease score (within 5 points’ range).

### Data abstraction

After matching was performed, patient charts were retrospectively reviewed from the date of LT. Baseline variables were abstracted at the time of LT. Outcomes of interest included incidence of biliary strictures, bile leak, stricture recurrence rate, number of interventions required for stricture resolution, and death. Bile leak was defined as biliary drainage, either from an abdominal drain or biloma noted on cross-sectional abdominal imaging. Biliary strictures were defined as anastomotic stenosis, preventing effective drainage of contrast material on cholangiography, requiring serial stent placement or biliary dilatation via ERCP or percutaneous transhepatic cholangiography (PTC) until complete resolution. ERCP was typically attempted first; if not feasible, PTC was then attempted. If the stenosis was not significant enough to interfere with the contrast flow during cholangiography, it was then excluded from the analysis and not considered a clinically significant biliary stricture.

Early biliary strictures were defined as those occurring within the first 3 months of transplant. Late biliary strictures occurred 3 months after LT. Non-anastomotic strictures, defined as strictures located >5 mm from the anastomotic site, were excluded from the analysis. Recurrence of biliary strictures was defined as re-development of anastomotic stenosis after complete resolution documented via an ERCP or PTC. These definitions were based off previously described literature regarding endoscopic treatment of anastomotic strictures.^7^, ^8^, ^9^

### Endoscopic protocol

All endoscopic procedures were performed after written informed consent was provided. Following an overnight fast and one dose of prophylactic antibiotics (2 g of ampicillin and 2 g of cefazolin), ERCP was performed. After selective wire-guided biliary cannulation, the biliary anastomosis was evaluated by cholangiography. The anastomotic biliary stricture was dilated by using a balloon dilator (Hurricane RX; Boston Scientific, Marlborough, Mass, USA) and a plastic biliary stent (Advanix [Boston Scientific] or Johlin [Cook Medical, Bloomington, Ind, USA]) was placed; choice of stent was at the endoscopist’s discretion. The diameter of the biliary stent was based on the lumen size of the donor bile duct. Follow-up ERCPs were then performed every 2 to 3 months per protocol or earlier if needed due to development of acute cholangitis or obstructive jaundice or if the stent became dislodged. During follow-up, the previous stent was removed and the degree of residual stenosis evaluated. Side-by-side stenting was commonly used on follow-up ERCPs until there was documented resolution of the stenosis across the anastomosis on cholangiography.

### Statistical analysis

Statistical analysis was performed by using statistical software R 4.0.4 (R Foundation for Statistical Computing, Vienna, Austria). Categorical data are expressed as number (%), and continuous variables are expressed as median and interquartile range, unless otherwise stated. Categorical variables were compared by using the χ^2^ test if the sample size was >50 and the Fisher exact test if the sample size was <50. Continuous variables were compared by using the nonparametric Mann-Whitney test. If the sample size was <50, the Fisher exact test was used to compare categorical variables. Statistical significance was determined by a *P* value <.05. Subjects were followed up from the time of LT (baseline) until last follow-up or death. Censoring occurred at the time of last follow-up or death. Cox proportional hazards regression was used to identify the association between different covariates and the development of biliary strictures. Results are expressed as hazard ratio and 95% confidence interval. To prevent instability in the model given the limited number of events, only 2 variables were allowed into the multivariable analyses.

## Results

### Baseline demographic characteristics

A total of 50 consecutive patients had LDLT with duct-to-duct anastomosis at our institution between December 11, 2011, and July 1, 2020. The characteristics of the 50 patients are presented in Table 1. The most common reason for LDLT was hepatocellular carcinoma (42%). Patients who underwent LDLT were followed up for a longer time than those who underwent DDLT (4.87 [2.36-6.40] years and 2.85 [1.98-4.37] years, respectively; *P* = .002). All transplants were from deceased brain death donors.

**Table 1:** Baseline characteristics

Variable	LDLT (n = 50)	DDLT (n = 50)
Age, y	58.00 (52.00-65.50)	58.50 (54.89-61.15)
Female sex	21 (43%)	21 (43%)
MELD score (average)	17	15
Cold ischemia time, min∗	41.50 (30.50-66.50)	370.00 (297.00-428.00)
Multi-biliary duct anastomosis	8 (16%)	7 (14%)
Placement of intra-operative biliary stent	37 (74%)	41 (82%)
Indication for LT		
Hepatocellular carcinoma	21 (42%)	21 (42%)
Alcohol	5 (10%)	5 (10%)
Hepatitis C	5 (10%)	5 (10%)
Primary biliary cholangitis	8 (16%)	8 (16%)
Primary sclerosing cholangitis	2 (4%)	2 (4%)
Wilson’s disease	1 (2%)	1 (2%)
Autoimmune hepatitis	1 (2%)	1 (2%)
Cryptogenic	2 (4%)	2 (4%)
NASH	3 (6%)	3 (6%)
Polycystic disease	1 (2%)	1 (2%)
Alpha_1_-antitrypsin deficiency	1 (2%)	1 (2%)

Values are median (interquartile range) unless otherwise indicated.

*LDLT*, Living donor liver transplantation; *DDLT*, deceased donor liver transplantation; *MELD*, Model for End-Stage Liver Disease; *LT*, liver transplantation; *NASH*, nonalcoholic steatohepatitis.

∗ Cold ischemia time was significantly shorter in the LDLT group compared with the DDLT group (*P* < .001). None of the other variables were statistically significant between the LDLT and DDLT groups (*P* > .05).

### Biliary leaks after LDLT and DDLT

There was a higher proportion of patients with biliary leak in the LDLT cohort compared with the DDLT cohort (14 [28%] vs 5 [10%], respectively; *P* < .01) (Table 2). Among the 14 patients with LDLT who developed bile leak, 6 were treated conservatively, resolving without PTC or ERCP; 2 had up-front biliary drainage (1 with ERCP and 1 with PTC); and 6 eventually developed anastomotic stricture. Among 5 patients with DDLT who developed bile leak, 4 were treated conservatively, and 1 was treated with up-front ERCP due to jaundice; a total of 2 patients eventually developed anastomotic stricture.

**Table 2:** Biliary adverse events

Variable	LDLT	DDLT	*P* value
Biliary stricture	14 (28%)	16 (32%)	.68
Bile leak	14 (28%)	5 (10%)	.01
Median time to stricture, mo	4.5	2	.06
Stricture recurrence	3 (6%)	3 (6%)	1
Median time to resolution (ERCP), mo	6.5	4	.24
Median no. of interventions (ERCP)	3	4	.79

*LDLT*, Living donor liver transplantation; *DDLT*, deceased donor liver transplantation.

### Biliary strictures after LDLT and DDLT

The proportion of biliary strictures was similar in both the LDLT and DDLT groups (14 [28%] vs 15 [30%] patients, respectively; *P* = .68). The median time to stricture formation was lower in the DDLT group (2 months) versus the LDLT group (4.5 months), although this difference did not reach statistical significance (*P* = .06). The proportion of biliary strictures in patients who did not have an intra-operative biliary stent placed during transplant was similar in both the LDLT and DDLT groups (6 of 13 [46.15%] vs 2 of 9 [22.22%] patients; *P* = .38). Four patients in the LDLT group had developed early biliary strictures, whereas 10 patients in the DDLT group had developed early biliary strictures (*P* = .09). The type of LT was not associated with the development of biliary strictures in the multivariable regression analysis (hazard ratio, 1.41; 95% confidence interval, 0.19-10.23; *P* = .74) (Table 3).

**Table 3:** Univariable and multivariable analysis of prognostic factors associated with development of biliary strictures

Variable	Univariable analysis		Multivariable analysis	
	HR (95% CI)	*P* value	HR (95% CI)	*P* value
Age at time of transplant	.02 (.95-1.04)	.77		
Female	.90 (.43-1.90)	.79		
LDLT	.41 (.18-.92)	.03	1.41 (.19-10.23)	.74
Multi biliary duct anastomosis	2.01 (.88-4.59)	.10		
Cold ischemia time	1.00 (1.00-1.01)	.01	1.00 (1.00-1.01)	.13
Intra-operative biliary stenting	.59 (.26-1.36)	.21		

*HR*, Hazard ratio; *CI*, confidence interval.

There was no difference in the median number of interventions required to resolve the strictures in the LDLT group compared with the DDLT group (3 [2-6] vs 4 [3-5], respectively; *P* = .79]. Similarly, there was no difference in the time to resolution of stricture in either group (6.5 [5-9] vs 4 [3-5] months; *P* = .24). Three patients in each group had recurrence of stricture after resolution. Four patients in the LDLT group compared with 1 patient in the DDLT group required PTC for stricture treatment (*P* = .11). Mortality in patients was similar in both LDLT and DDLT groups (5 versus 6 patients, *P* = .75).

In the LDLT group, 11 patients (22%) required ERCP interventions (10 for stricture and 1 for bile leak), 3 patients required PTC interventions after failed ERCPs (failed deep biliary cannulation with inability to access donor duct with wire), and 1 patient required ERCP initially for 3 interventions but ultimately started receiving PTC interventions because of poor treatment response. Patients treated with ERCP had a similar time to resolution of stricture compared with PTC: 6.5 (5-9) months versus 6 (4.5-6.5) months (*P* = .23). However, fewer ERCPs were required compared with PTCs to achieve stricture resolution: 3 (2-6) interventions versus 9 (7-11.5) interventions (*P* = .03). Recurrence of biliary stricture occurred in 1 patient treated with ERCP and in 2 patients treated with PTC. In the DDLT group, 15 patients required biliary interventions for either stricture or leak, of whom only 1 patient required PTC due to failed deep biliary cannulation (inability to access donor duct with wire). We did not compare ERCP versus PTC in DDLT patients given that only 1 patient required PTC.

## Discussion

Biliary adverse events are frequently encountered after an LT, with leaks and strictures occurring in anywhere from 10% to 45% of patients.^9^, ^10^, ^11^ Anastomotic biliary strictures have been thought to occur more frequently in LDLT patients compared with DDLT patients.^9^ Our study showed that LDLT was not associated with higher rates of biliary stricture compared with DDLT. In addition, we found that both the number of interventions as well as the time to resolution were similar in the LDLT group compared with the DDLT group. In LDLT patients who did require PTC, a higher number of interventions was typically required. Furthermore, the presence of biliary adverse events in patients who received LDLT was not associated with higher mortality rates.^12^ None of the patients in our study required re-transplantation or Roux-en-Y hepaticojejunostomy formation for recalcitrant strictures, although this has previously been described.^13^

Some studies have shown a higher rate of anastomotic biliary strictures in LDLT patients compared with DDLT patients.^9^ The reasons for this are multifactorial, including the fact that 2 anastomoses are often required, there is a more frequent duct-to-duct size mismatch, and an increased incidence of bile leak may occur.^12^ Although we did not show a higher rate of biliary strictures, our incidence of anastomotic biliary strictures in LDLT was similar to those reported in the literature. Han et al^14^ recently reported a 29.3% incidence rate of biliary strictures in 810 patients who had undergone an LDLT over the span of 14 years. Furthermore, our study mirrors prior reports of higher incidence of bile leaks in LDLT, with an overall incidence similar to what we reported.^15^^,^^16^

The therapeutic role of ERCP in managing anastomotic biliary strictures is clearly defined.^17^^,^^18^ Interestingly, the number of interventions needed to manage the biliary strictures in the LDLT was lower when using ERCP over PTC. We could not further evaluate outcomes between ERCP and PTC because of the relatively small sample size. Lee et al^19^ evaluated outcomes of using ERCP versus PTC in biliary strictures after LT; they found a 3-fold higher number of interventions needed using PTC compared with ERCP to resolve the strictures, which is similar to what the current study found. Other institutions have shown that, even in patients with Roux-en-Y hepaticojejunostomy biliary reconstruction, ERCP via enteroscopy resulted in fewer procedures compared with PTC for therapy of anastomotic strictures.^20^

The current study has multiple limitations. First, this was a retrospective, single-center study, which may limit the generalizability; despite our rigorous matching, it is still subject to selection bias. Second, our results are limited by the relatively small sample size in our study, although this may reflect the underutilization and lack of enthusiasm regarding use of duct-to-duct anastomosis in LDLT, perhaps due to fear of biliary adverse events.^21^, ^22^, ^23^ Third, we did not assess for donor adverse events, which are often a leading reason for the hesitation regarding more frequent utilization of LDLT in the United States.^3^ Fourth, we did not conduct a head-to-head comparison between ERCP and PTC owing to the small sample size, as mentioned earlier. Finally, none of our patients had fully covered self-expandable metal stents placed, although the efficacy of these stents has been previously described.^24^^,^^25^

In conclusion, the current study is one of the larger studies comparing biliary adverse event rates in LDLT versus DDLT in the United States. We found that patients who undergo duct-to-duct LDLT at a high-volume center did not have associated higher rates of biliary strictures. In addition, with experienced therapeutic endoscopists, patients who go on to develop strictures after LDLT did just as well as their counterparts with DDLT. The rate of biliary leaks remains significantly higher in LDLT compared with DDLT. However, there was no difference in mortality between groups. Duct-to-duct anastomosis LDLT should be frequently considered in patients with end-stage liver disease with favorable anatomy.

## Disclosure

All authors disclosed no financial relationships.
